# Random Processes Underlie Most Evolutionary Changes in Gene Expression

**DOI:** 10.1371/journal.pbio.0020154

**Published:** 2004-05-11

## Abstract

xx

Are evolutionary changes in gene expression determined mostly by natural selection or by random forces? It's been some 150 years since Charles Darwin proposed that organisms adapt to their environment through the process of natural selection, yet the debate still rages, particularly at the molecular level. Darwinian selection was challenged in 1983 by the Kimura neutral theory of molecular evolution, which argues that the majority of differences in DNA (nucleotide) and protein (amino acid) sequences within and between species have only minor or no selective effect and that these differences arise through mostly random processes. Mutations at the nucleotide level occur randomly and regularly. Some of them survive through generations, resulting in “fixed” evolutionary changes between species. Two potential mechanisms can lead to the fixation of a particular change: natural selection, which favors changes that convey a selective advantage, and stochastic (random) events, such as genetic drift (the random fluctuations in genotype frequencies that occur from generation to generation in small populations).[Fig pbio-0020154-g001]

**Figure pbio-0020154-g001:**
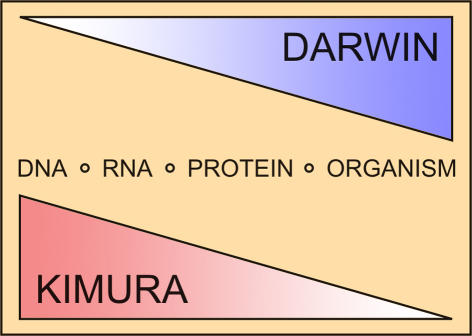


DNA mutations can lead to changes in gene expression levels, some of which may convey a selective advantage to an organism and therefore become fixed via natural selection. But since variation is produced at the genotype level, while selection is thought to operate largely at the phenotype level (that is, the physical manifestation of the genotype), it is reasonable to expect selection to be less apparent at the level of DNA sequence, and by extension, at the level of gene expression. Microarray technology has made it possible to systematically study expression levels of thousands of transcripts (the RNA copies of DNA that are translated into amino acid sequences) and to ask whether most changes of gene expression fixed during evolution between species result from selective or stochastic processes.

To investigate this question, Philipp Khaitovich and colleagues analyzed the observed transcriptome differences among primate and mouse species as well as among various brain regions within a species. The team started out by analyzing the expression levels of some 12,000 genes in the prefrontal cortex of various primates, including humans. If evolutionary changes are caused by chance and not by natural selection, they will accumulate as a function of time rather than as a function of physical or behavioral changes in the organism. And that's what the authors found: the changes in gene expression among the species progressed linearly with time, suggesting that gene expression in primate brains evolved in large part from random processes introducing selectively neutral, or biologically insignificant, changes.

According to neutral evolution theory, the same forces determine the rate of evolution both within and between species because similar random processes are at work on both levels. Consequently, genes that vary more within species should be more likely to vary between species. Comparing the expression levels of genes according to their variation within humans, the authors showed that genes with high variation among humans changed significantly faster between species than genes with low variation among humans. The authors also compared changes observed in genes to changes observed in pseudogenes (genes that over evolutionary time acquire a mutation that renders them nonfunctional) and found no significant difference between the two, suggesting again that most expression changes have no functional significance.

While their analysis cannot exclude a role for natural selection, all the results are consistent with a neutral model of transcriptome evolution. This means that the majority of gene expression differences within and between species are not functional adaptations but selectively neutral and that we won't be able to explain species differences based on variation in gene expression in general.

In addition to examining differences in gene expression in a particular tissue between species, the authors also discuss the evolution of different tissues within a species. The human brain is composed of regions that differ in function and histology (microscopic structure). Each of these regions acquired a functional or histological difference that separated it from its sister regions at some point in our evolutionary past. The authors show that the amount of change between regions correlates with tissue-divergence times estimated by other methods. If this finding applies for other tissues within and outside the brain, it could provide a method to reconstruct the evolution of tissues within a species.

